# Drinking and Death

**Published:** 1880-09

**Authors:** 


### "DRINKING" AND DEATH

Of soul, body and estate, how certain, soon and swift the sequence. R. M. H ———., who for a full quarter of a century has been an untiring minister of good to the sick and poor among us says that, half of all the city charities are swallowed up in removing the ills occasioned by intemperance.

We believe that if the sale of liquor of every name could be prevented, from sunset to sunrise, of every day, and for the whole of Sunday, it would be a direct annual saving of a million of dollars to the city treasury; and that crime, from petty theft to deliberate murder, would be prevented every year, aye, every month! enough to make an angel weep for joy. All honor to the men who are giving their time, their influence, their money, and more than all, their personal labor, to break up "The Sunday Liquor Traffic." It is a nobler effort than to win a battle, or found an empire, for it will be if successful, the saving of men enough to people an empire, every year!

